# Mapping the Bibliometrics Landscape of AI in Medicine: Methodological Study

**DOI:** 10.2196/45815

**Published:** 2023-12-08

**Authors:** Jin Shi, David Bendig, Horst Christian Vollmar, Peter Rasche

**Affiliations:** 1 Institute for Entrepreneurship University of Münster Münster Germany; 2 Institute of General Practice and Family Medicine Ruhr University Bochum Bochum Germany; 3 Department of Healthcare University of Applied Science - Hochschule Niederrhein Krefeld Germany

**Keywords:** artificial intelligence, AI, AI in medicine, medical AI taxonomy, Python, latent Dirichlet allocation, LDA, topic modeling, unsupervised machine learning

## Abstract

**Background:**

Artificial intelligence (AI), conceived in the 1950s, has permeated numerous industries, intensifying in tandem with advancements in computing power. Despite the widespread adoption of AI, its integration into medicine trails other sectors. However, medical AI research has experienced substantial growth, attracting considerable attention from researchers and practitioners.

**Objective:**

In the absence of an existing framework, this study aims to outline the current landscape of medical AI research and provide insights into its future developments by examining all AI-related studies within PubMed over the past 2 decades. We also propose potential data acquisition and analysis methods, developed using Python (version 3.11) and to be executed in Spyder IDE (version 5.4.3), for future analogous research.

**Methods:**

Our dual-pronged approach involved (1) retrieving publication metadata related to AI from PubMed (spanning 2000-2022) via Python, including titles, abstracts, authors, journals, country, and publishing years, followed by keyword frequency analysis and (2) classifying relevant topics using latent Dirichlet allocation, an unsupervised machine learning approach, and defining the research scope of AI in medicine. In the absence of a universal medical AI taxonomy, we used an AI dictionary based on the European Commission Joint Research Centre AI Watch report, which emphasizes 8 domains: reasoning, planning, learning, perception, communication, integration and interaction, service, and AI ethics and philosophy.

**Results:**

From 2000 to 2022, a comprehensive analysis of 307,701 AI-related publications from PubMed highlighted a 36-fold increase. The United States emerged as a clear frontrunner, producing 68,502 of these articles. Despite its substantial contribution in terms of volume, China lagged in terms of citation impact. Diving into specific AI domains, as the Joint Research Centre AI Watch report categorized, the learning domain emerged dominant. Our classification analysis meticulously traced the nuanced research trajectories across each domain, revealing the multifaceted and evolving nature of AI’s application in the realm of medicine.

**Conclusions:**

The research topics have evolved as the volume of AI studies increases annually. Machine learning remains central to medical AI research, with deep learning expected to maintain its fundamental role. Empowered by predictive algorithms, pattern recognition, and imaging analysis capabilities, the future of AI research in medicine is anticipated to concentrate on medical diagnosis, robotic intervention, and disease management. Our topic modeling outcomes provide a clear insight into the focus of AI research in medicine over the past decades and lay the groundwork for predicting future directions. The domains that have attracted considerable research attention, primarily the learning domain, will continue to shape the trajectory of AI in medicine. Given the observed growing interest, the domain of AI ethics and philosophy also stands out as a prospective area of increased focus.

## Introduction

### Background

Artificial intelligence (AI) evolved from its conceptual inception in the 1950s [1-4] to being a transformative force across various sectors [5]. However, its penetration into the medical arena has been comparatively slower, constrained by factors such as escalating costs, rigorous regulations, and exacting performance standards [6-9]. Regardless of these hurdles, the promise of AI in reshaping and individualizing health care has never been more evident [5,9,10]. Its potential for revolutionizing medical practices, enhancing patient care quality, and improving health care efficiency has spurred substantial research endeavors [10-14], with recent years witnessing an unprecedented surge in medical AI studies [3,15,16].

However, amidst this burgeoning research, a clear consensus on AI’s scope and definition within medicine remains elusive [9,17]. Although Meskó and Görög [14] detailed 3 AI levels and Hamet and Tremblay [18] bifurcated AI into virtual and physical domains, the multifaceted nature of AI continues to demand diverse considerations, depending on the context [19]. Further complicating the landscape is AI’s application in various medical niches, such as mental illness diagnosis [20], pandemic readiness [21], or areas such as endoscopic imaging [16] and acute stroke treatments [22].

Despite such vast explorations, the AI research landscape has not been immune to challenges. The well-documented AI winters [1], periods of waning interest, starkly contrast the consistent ascent of medical AI studies in more recent times [14]. This brings us to an essential question: How has the landscape of AI in medicine morphed over time? Is there a comprehensive understanding of its trajectory and a detailed mapping of its vast applications?

### Aim of the Research

To address this knowledge gap, our study endeavors to provide a systematic, temporal assessment. We conducted a bibliometric exploration spanning 23 years, harnessing data from publications indexed in PubMed. The intent is 2-fold: to offer a comprehensive overview of the progression of medical AI and to discern emerging patterns and prospective directions. In doing so, we aim to fortify the foundational understanding of AI in medicine, setting the stage for subsequent in-depth explorations.

## Methods

### Overview

The main challenge of this study involves collating a vast array of research in a comprehensive yet succinct manner. To this end, we used a computer-aided bibliometrics analysis using Python (version 3.11) and the Spyder IDE (version 5.4.3), capitalizing on Python’s capacity to aggregate and analyze medical AI articles indexed in PubMed since 2000. Using this approach, we systematically parsed the keywords from PubMed’s AI publication to identify patterns and focal points in medical AI research.

Our methodology incorporates a computer-aided text analysis within the framework of bibliometric analysis, a technique increasingly favored across various disciplines, particularly in medicine, because of its ability to interpret semantic meaning [23]. Key to our analysis was text mining and unsupervised machine learning topic modeling facilitated by a Python algorithm, methods known to offer critical insights into existing studies and future research directions [24].

### Study Period

Our study spanned from 2000 to 2022, a more extensive timeframe than previous literature reviews. In this 23-year duration, we extracted a substantial number of articles from PubMed, justifying our decision to use a computational analysis approach, given the anticipated vast search results.

### Keywords Identification Strategy

#### Two-Pronged Search Strategy

Previous research often used a broad concept of [17,19] conducting the literature reviews based on specific keywords, such as machine learning or deep learning [14,25]. However, this approach overlooks numerous AI-related articles. Given the absence of a predefined framework for this analysis, it was critical to identify search keywords. Thus, our first crucial task involved a 2-pronged search strategy: using Medical Subject Headings (MeSH) terms and associated text keywords derived from those MeSH terms. This methodology was adopted to ensure an expansive capture of medical AI articles. The detailed search strategy is presented in Multimedia Appendix 1 [14,17,19,26-30]. For example, when searching for articles on the topic of deep learning, our Python script was designed to perform a comprehensive scan. This involved not only searching for the MeSH tag *deep learning* but also using *deep learning* as a text search keyword in both the title and abstract. In addition, we incorporated the entry terms associated with the *deep learning* MeSH tag into our search criteria. This search strategy has been shown to enhance the efficiency of the literature review [31].

#### Distribution Analysis

Upon obtaining the search results using this method, we took the analysis a step further by investigating the distribution of research across various AI domains. To accomplish this, we devised a dictionary based on the 8 AI domains and associated keywords identified in the 2020 AI Watch report published by the European Commission Joint Research Centre (JRC) [17] (Textbox 1). This dictionary served as our analytical tool, helping us discern and understand the evolving research patterns in the diverse AI sector based on our search results from PubMed.

**Artificial intelligence (AI) domains from the Joint Research Centre AI Watch report.**

**AI domain and subdomains**

- Core
  Reasoning
    Knowledge representation
    Automated reasoning
    Common sense reasoning
  Planning
    Planning and scheduling
    Searching
    Optimization
  Learning
    Machine learning
  Communication
    Natural language processing
  Perception
    Computer vision
    Audio processing
- Transversal
  Integration and interaction
    Multiagent systems
    Robotics and automation
    Connected and automated vehicles
  Service
    AI services
  AI ethics and philosophy
    AI ethics
    Philosophy of AI

### Eligibility Criteria for Article Selection

Articles that fulfilled the following criteria were included in this study: (1) articles with artificial intelligence MeSH tags or AI-related keywords in the titles and abstracts; (2) articles published from January 1, 2000, to December 31, 2022, in PubMed; (3) articles written in English; and (4) peer-reviewed journal articles. The abovementioned 4 searching criteria were coded directly in our Python searching script.

### Data Processing

**Figure 1:**
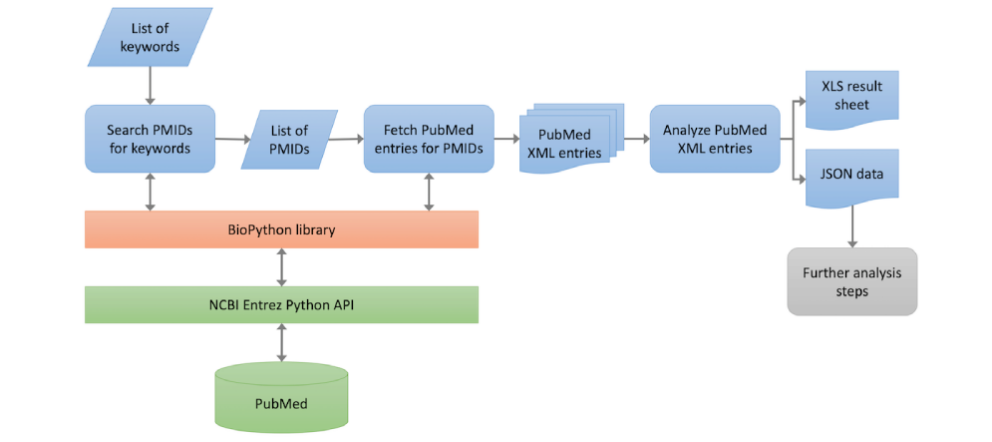
Flow chart of data processing with Python from PubMed. A list of artificial intelligence keywords were searched in PubMed. Publication that contained the keywords were fetched, and metadata, including article titles, abstract, author names, journal names, and publication date, were saved in a datasheet.

Given the restrictions imposed by PubMed on the volume of downloadable publication data, our approach required the use of a custom Python algorithm. This algorithm efficiently retrieved extensive metadata from articles regarding AI in medicine, encompassing the article titles, authors, publication dates, and abstracts. The application of Python allowed us to bypass these limitations and ensure a comprehensive collection of relevant data. Figure 1 illustrates the process we carried out using Python to extract data from PubMed.

Concurrently, within the structure of computer-aided text analysis, our Python algorithm directly counted keyword frequencies in the titles and abstracts from the acquired datasheet while assigning the relevant articles to their respective domains based on the JRC AI Watch report.

### Topic Modeling Using Latent Dirichlet Allocation

Drawing from the metadata of publications retrieved from PubMed, we used latent Dirichlet allocation (LDA) topic modeling—an established method in various academic research fields, including technology management, computer science, and biomedicine [32-34]—to discern shifts in research areas within individual AI domains. Our LDA topic modeling, conducted in Python using the Gensim library, used unsupervised machine learning to analyze vast quantities of unstructured data. It allocated each article to a probable topic based on word frequency [33,35].

The Gensim LDA model, premised on fundamental natural language processing concepts, initiated the process by cleaning the data and then preparing the tokens, corpus, and dictionary before training the program for topic clustering [36,37]. The versatility of this method extends beyond our study as it is applicable to various data sources and disciplines. For instance, Abd-Alrazaq et al [38] used a topic modeling approach to identify top concerns regarding COVID-19 based on posts on Twitter, whereas Lee and Kang [32] applied the same method to find the top 50 topics in the technology and innovation management studies from 11,693 articles published in top technology and innovation management journals.

## Results

### Publication Analysis

**Figure 2:**
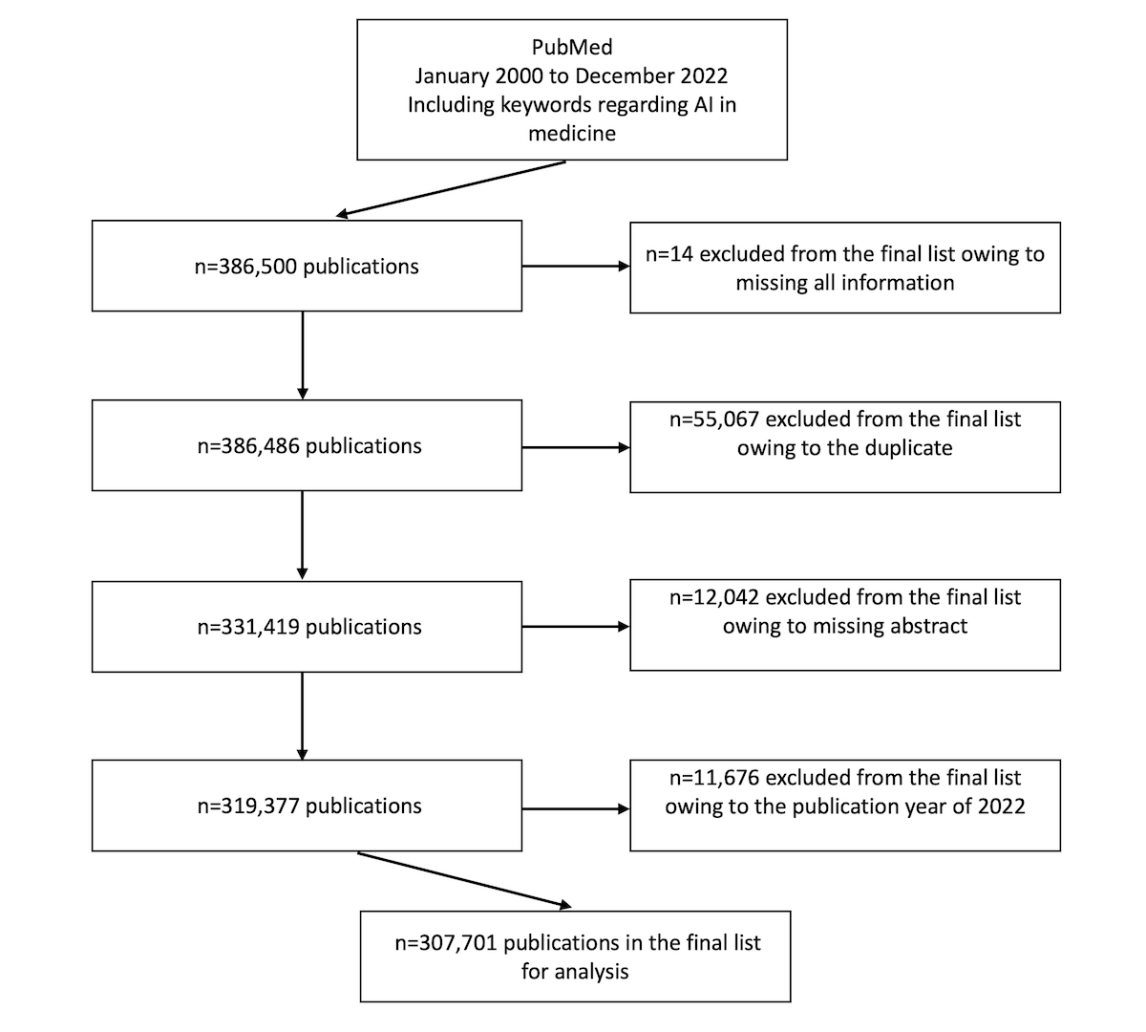
PubMed publications from 2000 to 2022 were screened and downloaded using medical artificial intelligence (AI) Medical Subject Headings terms and keywords.

**Figure 3:**
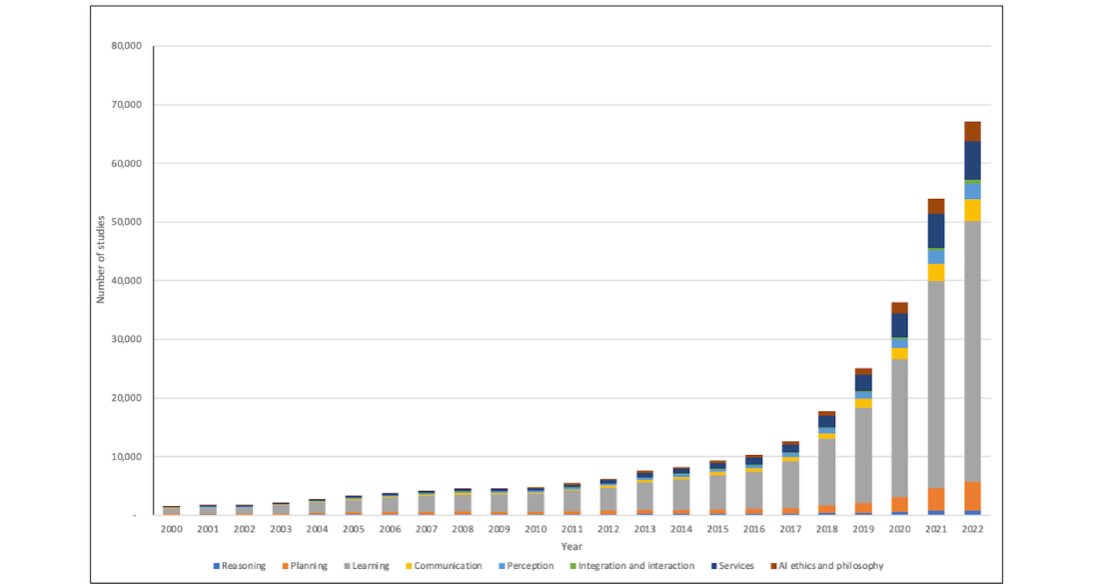
Number of artificial intelligence (AI) in medicine studies retrieved from PubMed by year from 2000 to 2022.

**Figure 4:**
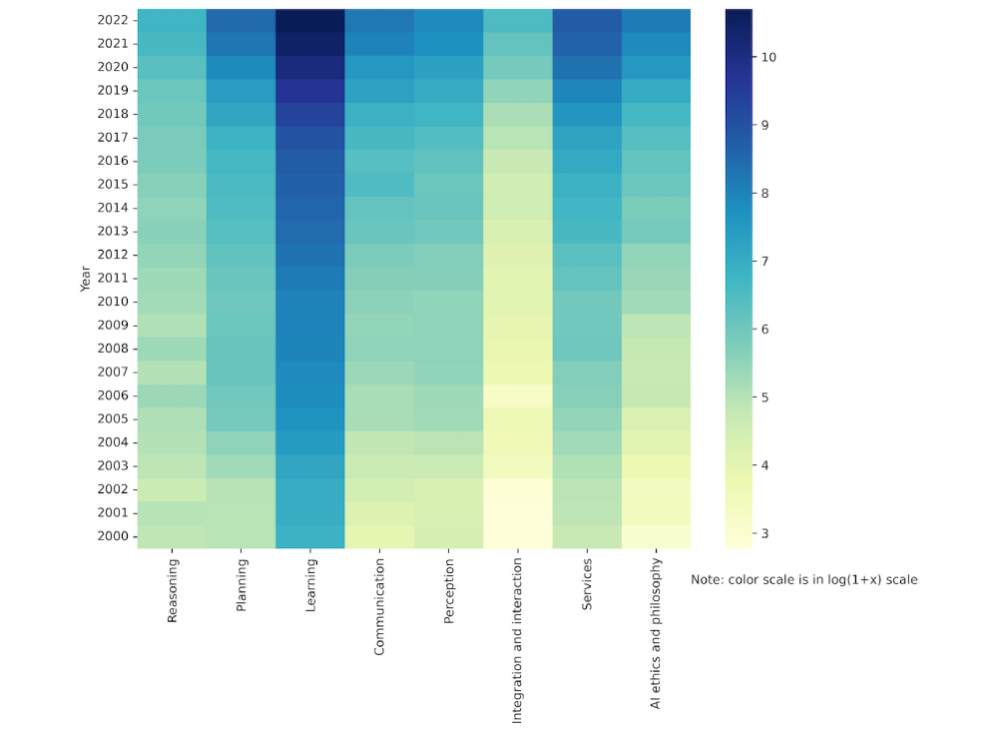
Publications in PubMed in log scale per domain per year from 2000 to 2022. AI: artificial intelligence.

To facilitate further analysis, we refined the search results by discarding publications that failed to meet our selection criteria, leaving us with a corpus of 307,701 entries (refer to Figure 2 for the selection process). Subsequently, these results were organized into 8 AI domains defined by JRC. PubMed’s AI-related publications have witnessed exponential growth, as detailed in Figure 3, and Figure 4 illustrates the distributions of studies across each domain over the years.

**Figure 5:**
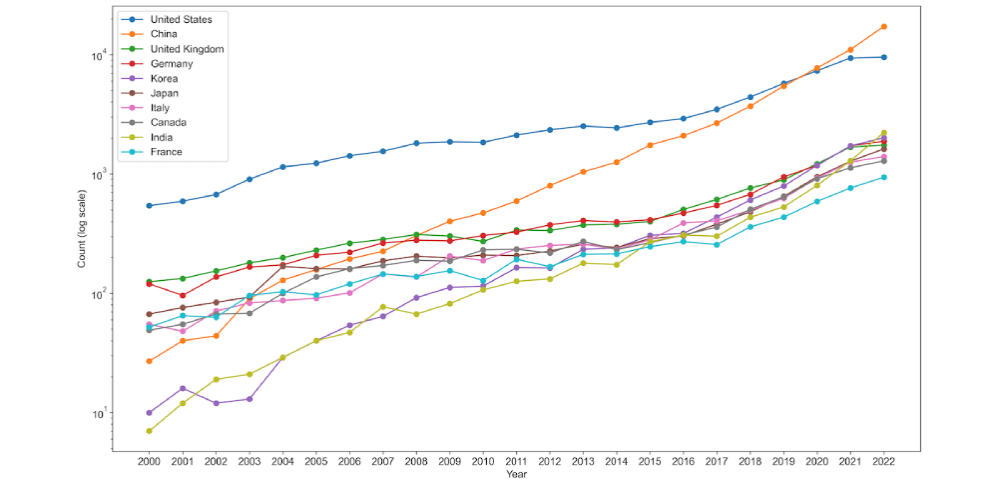
Annual artificial intelligence (AI)–related publication counts on PubMed by leading countries (2000-2022): nonaccumulative yearly data. Note: the chart uses a log scale for clarity owing to significant discrepancies in numbers, particularly from the United States and China.

The landscape of AI-related studies has undergone a substantial transformation over the past 2 decades. In 2000, we found just 1614 AI-related studies, a number that nearly quadrupled within a decade. By 2022, the count had surged to 58,458, representing a 36-fold increase. The geographic spread of these publications (Figure 5) shows the United States leading with 68,502 articles in the past 23 years, followed closely by China’s 57,460. Notably, China’s annual output of medical AI publications over the past 3 years has surpassed that of the United States. Our research strategy centered on the first author’s information to manage the complexity introduced by the considerable number of multiauthor publications. This decision allowed us to maintain the rigor of our analysis while navigating the vast data set effectively.

**Figure 6:**
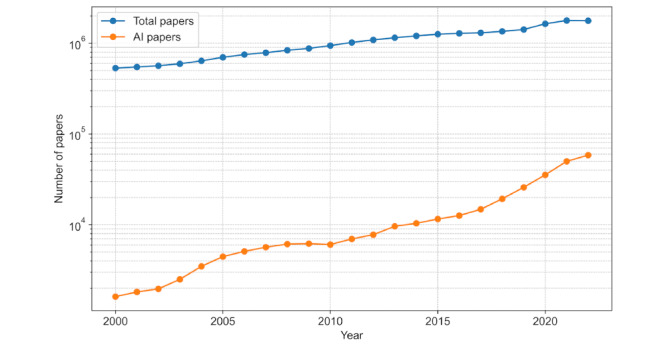
Number of total studies versus AI studies in PubMed (log-scale) by year from 2000 to 2022.

By juxtaposing the annual growth of AI publications with the total number of articles on PubMed (Figure 6), we found that although both increased annually, AI research grew more substantially. The learning domain stood out, contributing to 62.88% (16,254/25,850) to 76.09% (44,481/58,458) of AI research over the last 4 years and totaling 44,481 articles in 2022. An analysis of keyword occurrences within each domain reaffirmed this dominance; over half of the top 20 keywords belonged to the learning domain (Table 1).

**Table 1:** Top 20 keywords and their occurrence within investigated titles and abstracts.

Rank	Keywords	Frequency of appearance	Domain
1	“learning”	317,847	Learning
2	“machine learning”	133,891	Learning
3	“neural network”	130,111	Learning
4	“classification”	111,173	Learning
5	“deep learning”	78,044	Learning
6	“artificial intelligence”	38,918	General
7	“convolutional neural network”	30,180	Learning
8	“iot”	29,802	Service
9	“support vector machine”	28,792	Learning
10	“pattern recognition”	21,272	Learning
11	“optimization”	20,993	Planning
12	“artificial neural network”	20,306	Learning
13	“clustering”	17,828	Learning
14	“safety”	14,733	Artificial intelligence ethics and philosophy
15	“service”	14,409	Service
16	“communication”	12,151	Communication
17	“perception”	12,005	Perception
18	“planning”	10,601	Planning
19	“deep neural network”	10,384	Learning
20	“natural language processing”	9599	Communication

The communication, integration and interaction, and services domains have also grown, but their share remained relatively constant. Conversely, despite an annual increase in publication count, the reasoning domain lagged in overall growth. Research in AI ethics and the philosophy of AI, a relatively novel field, has shown promising growth, from 20 articles in 2000 to 2613 in 2021.

It should be noted that the sum of domain-specific articles in Table 2 does not match the total because of the different sets of keywords used for downloading articles (MeSH terms and associated text keywords) and counting words (JRC AI Watch report’s taxonomy). Similarly, the sum of domain-specific percentages in Table 2 does not add up to 100% because the articles overlap across domains, resulting in a sum >1 in specific years.

**Table 2:** The number and percentage of papers with artificial intelligence (AI) keywords (N=307,701).

Domain year	Reasoning, n (%)	Planning, n (%)	Learning, n (%)	Communication, n (%)	Perception, n (%)	Integration and interaction, n (%)	Services, n (%)	AI ethics and philosophy, n (%)	Total, n (%)
2000	130 (8.05)	140 (8.67)	938 (58.12)	53 (3.28)	75 (4.65)	15 (0.93)	115 (7.13)	20 (1.24)	1614 (0.52)
2001	147 (8.09)	140 (7.71)	1079 (59.38)	67 (3.69)	74 (4.07)	16 (0.88)	131 (7.21)	31 (1.71)	1817 (0.59)
2002	103 (5.24)	147 (7.48)	1109 (56.47)	86 (4.38)	74 (3.77)	17 (0.87)	135 (6.87)	30 (1.53)	1964 (0.64)
2003	132 (5.28)	200 (7.99)	1336 (53.40)	103 (4.12)	104 (4.16)	30 (1.20)	160 (6.39)	41 (1.64)	2502 (0.81)
2004	151 (4.34)	257 (7.39)	1757 (50.53)	125 (3.60)	137 (3.94)	35 (1.01)	197 (5.67)	57 (1.64)	3477 (1.13)
2005	165 (3.70)	367 (8.24)	2104 (47.24)	176 (3.95)	199 (4.47)	38 (0.85)	243 (5.46)	68 (1.53)	4454 (1.45)
2006	213 (4.19)	378 (7.43)	2445 (48.05)	175 (3.44)	202 (3.97)	25 (0.49)	283 (5.56)	116 (2.28)	5088 (1.65)
2007	149 (2.63)	465 (8.22)	2653 (46.90)	210 (3.71)	250 (4.42)	41 (0.72)	295 (5.21)	115 (2.03)	5657 (1.84)
2008	206 (3.36)	455 (7.43)	2892 (47.23)	253 (4.13)	253 (4.13)	45 (0.73)	405 (6.61)	117 (1.91)	6123 (1.99)
2009	159 (2.57)	436 (7.05)	3002 (48.54)	237 (3.83)	254 (4.11)	52 (0.84)	385 (6.23)	132 (2.13)	6184 (2.01)
2010	194 (3.21)	409 (6.76)	3076 (50.82)	262 (4.33)	251 (4.15)	57 (0.94)	377 (6.23)	197 (3.25)	6053 (1.97)
2011	205 (2.94)	450 (6.45)	3506 (50.27)	286 (4.10)	286 (4.10)	57 (0.82)	468 (6.71)	218 (3.13)	6975 (2.27)
2012	240 (3.09)	511 (6.57)	3986 (51.29)	331 (4.26)	300 (3.86)	63 (0.81)	538 (6.92)	237 (3.05)	7772 (2.53)
2013	274 (2.85)	599 (6.23)	4694 (48.81)	446 (4.64)	381 (3.96)	73 (0.76)	759 (7.89)	370 (3.85)	9617 (3.13)
2014	254 (2.45)	661 (6.37)	5155 (49.70)	477 (4.60)	444 (4.28)	90 (0.87)	826 (7.96)	331 (3.19)	10,372 (3.37)
2015	280 (2.42)	704 (6.09)	5804 (50.22)	658 (5.69)	429 (3.71)	92 (0.80)	971 (8.40)	426 (3.69)	11,558 (3.76)
2016	327 (2.59)	777 (6.16)	6349 (50.32)	593 (4.70)	503 (3.99)	109 (0.86)	1188 (9.42)	487 (3.86)	12,617 (4.10)
2017	342 (2.31)	901 (6.09)	7956 (53.76)	765 (5.17)	625 (4.22)	143 (0.97)	1344 (9.08)	594 (4.01)	14,799 (4.81)
2018	393 (2.03)	1333 (6.90)	11,272 (58.31)	974 (5.04)	843 (4.36)	172 (0.89)	1985 (10.27)	771 (3.99)	19,330 (6.28)
2019	427 (1.65)	1680 (6.50)	16,254 (62.88)	1461 (5.65)	1126 (4.36)	251 (0.97)	2773 (10.73)	1125 (4.35)	25,850 (8.40)
2020	567 (1.60)	2529 (7.13)	23,493 (66.24)	1884 (5.31)	1512 (4.26)	373 (1.05)	4119 (11.61)	1831 (5.16)	35,467 (11.53)
2021	749 (1.50)	3877 (7.76)	35,333 (70.73)	2938 (5.88)	2229 (4.46)	482 (0.96)	5741 (11.49)	2613 (5.23)	49,953 (16.23)
2022	845 (1.45)	4913 (8.40)	44,481 (76.09)	3667 (6.27)	2630 (4.50)	691 (1.18)	6516 (11.15)	3413 (5.84)	58,458 (19.00)
All years	6652 (2.16)	22,329 (7.26)	190,674 (61.97)	16,227 (5.27)	13,181 (4.28)	2967 (0.96)	29,954 (9.73)	13,340 (4.34)	307,701 (100)

### Citation Analysis

Given the sizable research data, conventional software such as VOSviewer (version 1.6.19; Leiden University) fell short of our needs. Hence, we crafted a Python script to extract author and citation data. From the pool of 307,701 publications, we found 1,054,040 contributing authors. Solo-author publications constitute a mere 3.69% (11,347/307,701), which dwindled from 13.44% (217/1614) in 2000 to 2.71% (1586/58,458) by 2022. This observation underscores an increasing inclination toward collaboration in medical AI research, likely propelled by the field’s complexity and advancement in technology. The collaboration index, which stood at 3.43, supports this finding. Given our broad perspective, we omitted h-index and i-index calculations because they offer limited insight when considering our extensive data set.

Over the past 23 years, the number of citations has reached 3,425,831, averaging 11 (SD 62.50) per publication. Regarding citations for individual countries, the United States leads by a considerable margin, boasting 1,196,517 citations with an average of 17 (SD 118.85) citations per article, far surpassing other nations. Despite China’s substantial publication count, its average number of citations per paper stood at 7 (SD 19.34), trailing behind the United States and several European countries. Table 3 illustrates the publications and citations of the top 10 active countries and organizations in the United States and China. Among these, Stanford University (United States) leads with an average of 41 (SD 484.08) citations per paper, whereas Zhejiang University (China), despite being prolific, has an average citation count of only 7 (SD 35.17).

**Table 3:** Top 10 most active countries and organizations in the United States and China.

		Publication, n	Citation, n	Citation, mean (SD)
**Countries**				
	United States	68,555	1,196,517	17 (118.85)
	China	57,564	376,363	7 (19.34)
	United Kingdom	11,708	221,617	19 (99.28)
	Germany	11,600	175,069	15 (54.39)
	Korea	8743	67,366	8 (16.81)
	Japan	8504	94,597	11 (87.11)
	Italy	7976	82,069	10 (21.82)
	Canada	7787	106,596	14 (50.13)
	India	7306	44,957	6 (15.95)
	France	5816	71,738	12 (51.14)
**Organizations in the United States**				
	Stanford University	1427	59,211	41 (484.08)
	University of Michigan	1299	25,939	20 (95.64)
	University of Pennsylvania	1234	24,293	20 (61.26)
	University of Pittsburgh	1069	15,462	14 (34.38)
	University of Washington	1034	25,864	25 (135.62)
	Johns Hopkins University	994	15,675	16 (38.04)
	Massachusetts Institute of Technology	830	18,934	23 (48.92)
	University of Southern California	799	16,605	21 (91.88)
	Vanderbilt University	716	12,408	17 (52.20)
	University of Maryland	716	8251	12 (26.71)
**Organizations in China**				
	Zhejiang University	1903	13,921	7 (35.17)
	Shanghai Jiao Tong University	1280	8990	7 (12.97)
	Fudan University	1182	9266	8 (17.06)
	Sichuan University	1078	7222	7 (14.72)
	Central South University	1059	8696	8 (15.46)
	Nanjing University	1049	5616	5 (10.17)
	Peking University	1016	10,632	10 (69.90)
	Peking Union Medical College	929	5585	6 (13.71)
	Wuhan University	925	5731	6 (13.33)
	Shandong University	896	5663	6 (12.94)

**Figure 7:**
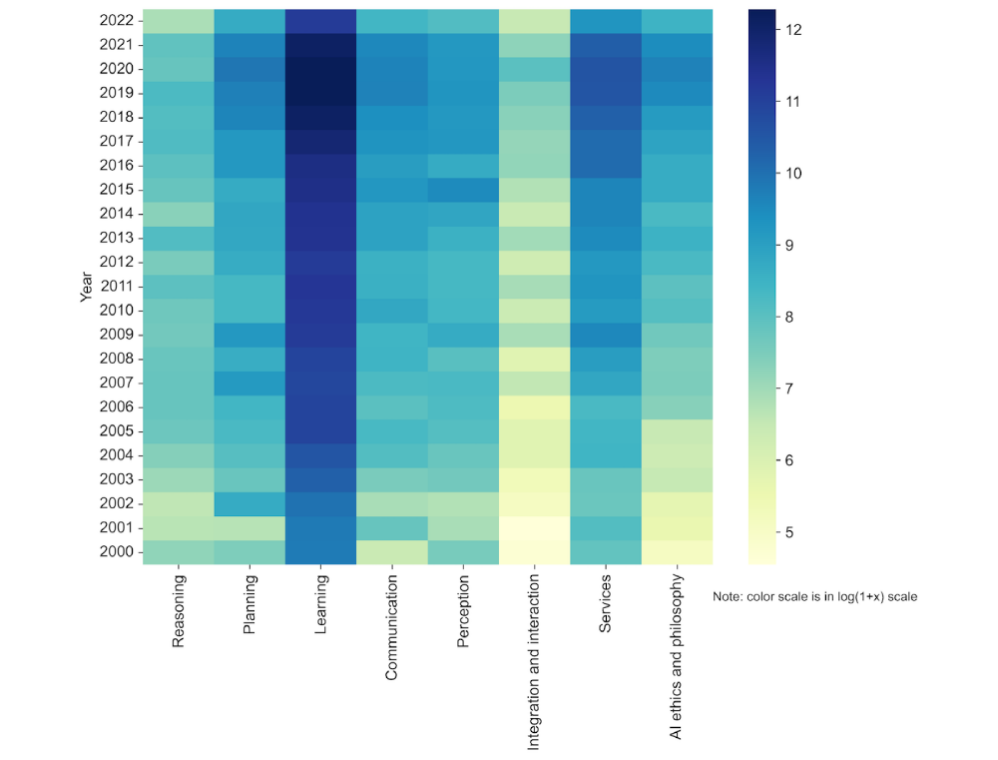
Citation number in PubMed in log scale per domain per year from 2000 to 2022. AI: artificial intelligence.

In the context of the JRC’s AI domains, the dominance of learning is also evident in its citation numbers. Figure 7 shows the citation situation across each domain. To account for the considerable citation disparity among domains, Figure 7 is presented in log scale, offering a more accurate picture of each domain’s standing.

### Science Mapping

In the span of the past 23 years, the most highly cited paper was published in 2003 and focused on an open software that helps scientists visualize and analyze how different molecules in a cell interact and can be customized with add-ons to perform even more specific studies [39]. Garnering 18,081 citations constitutes only 0.53% (18,081/3,425,831) of the total citation volume, demonstrating the breadth and diversity of the research within the field.

**Figure 8:**
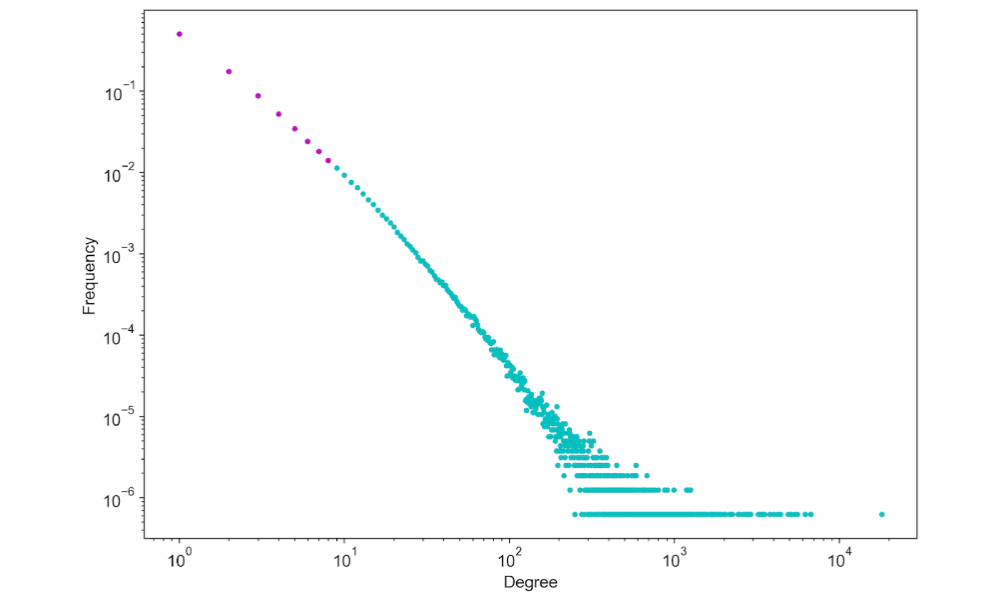
Degree distribution of citation network. This log-log plot represents the degree distribution of nodes within our citation network. Each dot corresponds to the number of papers (on the x-axis) with a certain number of citations (y-axis).

Through our Python-driven analysis, we comprehensively examined the degree distribution of publications and their respective citations, as depicted in Figure 8. This network comprises 1,603,481 nodes, representing individual papers, and 3,423,669 edges, symbolizing citations among these papers. Notably, many nodes have sparse connectivity, indicating papers with limited citations. However, a distinct subset, represented by the magenta dots, accounts for the top 1% of the papers with a remarkably high citation count. These papers, or “hubs,” act as the primary influencers in our network. Such a scale-free distribution, where a minority possesses numerous connections and the majority has fewer connections, mirrors common patterns in citation networks. This exemplifies the scenario where only a handful of papers garner the most citations.

Furthermore, our citation analysis revealed that only 1.21% (3716/307,701) of the papers had been cited >100 times. Astonishingly, a substantial 78.67% (242,054/307,701) had been cited <100 times, and 20.13% (61,931/307,701) had not been cited at all. On the basis of this discernment, we narrowed our subsequent analysis to focus exclusively on PubMed identifiers (PMIDs) with >100 citations, ensuring that we captured the most influential connections. By limiting the volume of papers in this manner, we were able to use VOSviewer effectively for illustrative visualizations of the data.

**Figure 9:**
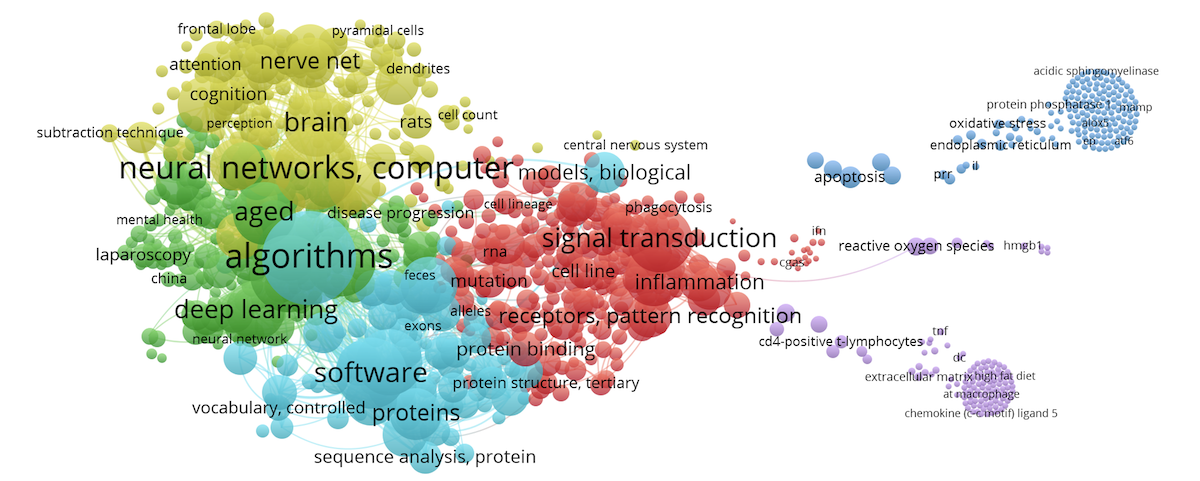
Distribution of themes.

**Figure 10:**
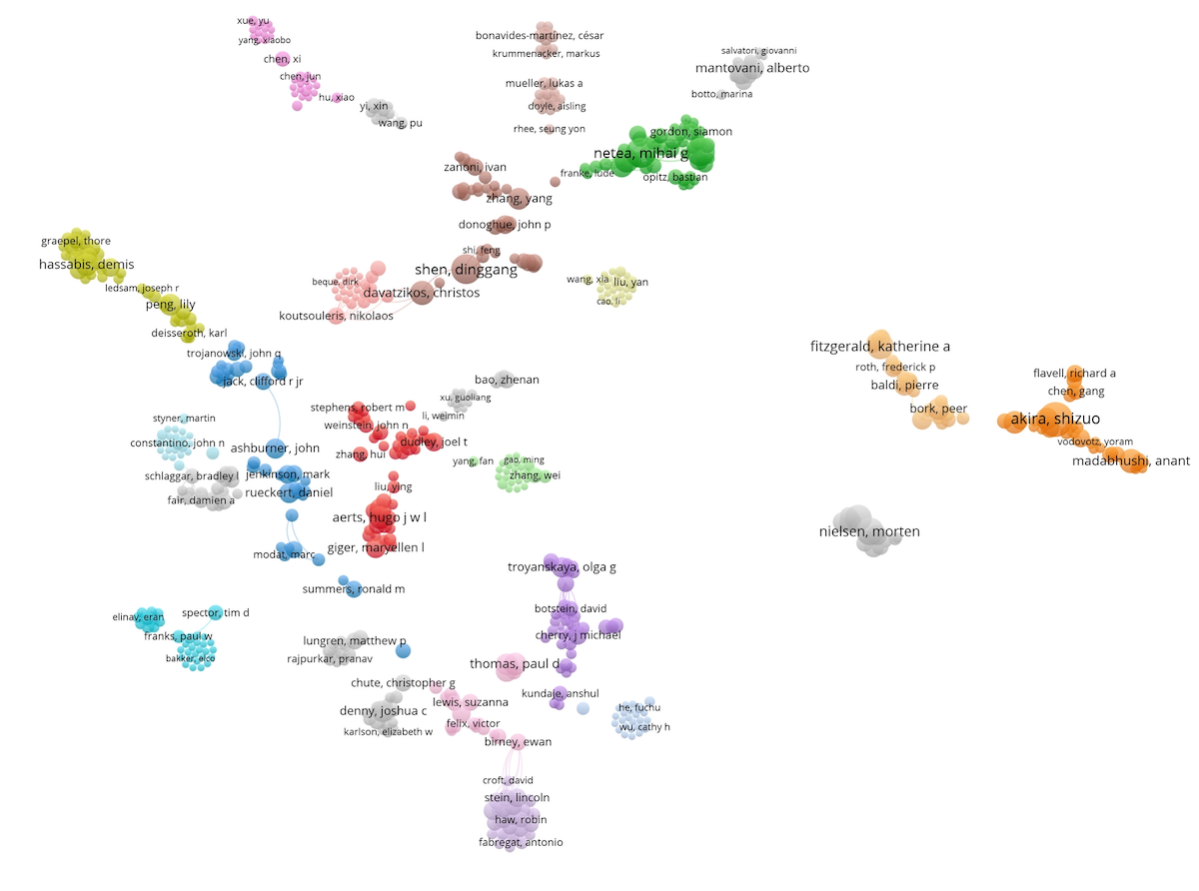
Coauthorship from the most cited paper in PubMed.

Figures 9 and 10 provide an intricate perspective on the network and coauthorship patterns of the most cited papers in PubMed [40]. Figure 9 portrays the core thematic clusters of AI research: red for signal transduction, yellow for neural networks, a meld of blue and green signifying algorithms, green representing deep learning, and blue demarcating software.

A salient observation from Figure 10 is the distinct sparsity of interconnections in the cocitation landscape among influential works. Although author names identify clusters, they align with specific thematic nuances, as suggested by our examination of the associated publications. The red cluster is associated with medical imaging [41,42], blue encompasses computational methods [43,44], brown touches on pattern recognition [43,45], lilac provides insights into genomics and genetics [46,47], and dark orange centers around immune response mechanisms [48,49]. The minimal connectivity among these clusters indicates less cross-domain collaboration than is typically observed [50,51]. Such an isolated pattern emphasizes the specialized nature of research, bolstering our decision to engage in detailed domain-specific topic modeling in our subsequent sections [52].

### Topic Analysis With Topic Modeling (LDA)

Although there are overarching themes in AI research in medicine, individual works seem to delve deeply into specific domains without broad interconnections with others. This siloed approach suggests that to genuinely understand the intricacies of medical AI, one must dive into each domain independently. Such insights form the foundation for our next analytical step. We harnessed the LDA technique by targeting the titles and abstracts of the entries from PubMed. This allowed us to tease out nuanced research topics from the vast data set. Given the expansive timeline and sheer volume of articles, we segmented the data into 5-year intervals, conducting distinct topic modeling for each period within separate AI domains. This strategic division enables a meticulous tracing of the progression of medical AI, offering a refined perspective on its multifaceted evolution.

Building on our observations that underscored the need for domain-specific exploration, our methodological choices in the succeeding phase took a meticulous approach. Owing to the surge in AI research publications, particularly in 2020 and 2021, we made an exception to group these years together for topic modeling. This was attributed to the substantially large volume of articles published during this brief period. Meanwhile, the notable output from 2022 was considered as an independent entity for the analysis.

By leveraging the capabilities of the LDA model, topics were extracted based on the keyword combinations identified by our Python algorithm. We associated these combinations with the PMIDs that best represented each topic within our Python script to make these combinations more interpretable. This aided in disambiguating the topics and ensured a deeper comprehension of the themes that sometimes seemed elusive owing to the abstract nature of the keyword groupings.

Our subsequent findings revealed exciting disparities in the volume of articles representing each topic. To streamline our results and accentuate the most impactful research areas, we arranged the topics according to the number of their corresponding articles. This allowed us to highlight the top 5 topics for each AI domain across the delineated time frames, as presented in Table 4.

**Table 4:** Top 5 topics in each domain.

AI^a^ domain		2000-2004		2005-2009		2010-2014		2015-2019		2020-2021	2022
**Core**											
	Reasoning	Diagnostic model, disease diagnosis, signal recognition, fuzzy control, and clinical knowledge	Ontology design pattern, fuzzy logic and medical treatment, medical diagnosis risk, gene database and fuzzy logic, and diagnostic knowledge		Medical decision, gene classification, fuzzy logic, baseline correction algorithm, and medical evidence		Clinical decision, biomedical ontology evaluation, robotic control assessment, protein and gene clustering and diagnosis, and diagnostic knowledge		Biomedical prediction, wearable sensor, clinical decision, imaging and clinical decision, and digital health control		Fuzzy logic assessment, machine learning analysis, data analysis, clinical diagnosis, and biomedical information assessment
	Planning	Image clustering, surgical assistance system, ANN^b^ and patient control, parameter descriptor and drug development, and experimental design	DNA sequence, graph matching problem, tumor segmentation, image training algorithm, and robotic surgery		Computer-aided detection and prediction, brain tumor segmentation, genetic algorithm, fuzzy neural network algorithm, and feature selection and classification		Drug disease response, image detection, object representation in the brain, protein in memory storage, and robotic surgery		Cell feature, 3D modeling, COVID -19 care, depression prediction, and MRI^c^ pattern		Robot control optimization, COVID-19 outcome, information enhancement, automatic search algorithm, and dynamic imaging reconstruction
	Learning	Mathematical computer models, protein classification, response prediction, statistical tool, and clinical imaging	Clinical imaging, fuzzy clustering, gene interaction networks, biomarker feature selection, and cancer prediction		Biomedicine image, gene classification, protein complex, ECG^d^ and chronic disease, and texture feature in diagnosis		Cancer cell and fuzzy rules, robotic surgery and disease prediction, video and object detection, brain imaging, and gene selection		MRI imaging and texture analysis, segmentation methods for medical images, imaging technology, ordered protein and region prediction, and sepsis and clinical care		Predictive medicine, structural biomarkers, disease diagnosis, convolutional radiology, and behavior recognition
	Communication	Communication in remote robotic surgery, medical semantic, imaging and semantics, NLP^e^ code, and gene expression	Gene expression, behavior and disease detection, protein annotation, image representation, and clinical document		Medical names extraction, protein complex detection, swarm robots searching, text mining, and semantic report		Ontology-based text mining, biomedical text, substance use in social media post, automatic clinic report annotation, and social narrative		COVID-19 and social media, drug abuse, drug control, biomedical named entity recognition, and cancer genetics		Electrical engineering in communication, machine learning evaluation, semantic biomedical applications, radiology platform control, and NLP in patient care
	Perception	Pattern recognition, sound recognition in auditory deficits, clinical system evaluation, image segmentation, and surface learning in preclinical years	Face processing in fMRI^f^, object recognition, feature recognition, pattern detection, and visual system		Cognitive simulation, image feature recognition, speech and pattern recognition, child care and brain development, and body sensor		Image clustering and gesture recognition, surgical task evaluation, image processing and imaging dose, robotic devices training, and face and language recognition		Activity recognition, object detection, sound recognition, tumor detection, and health care technology		Electronic information management, image retrieval, base model for detection and classification, neural network for speech processing, and clinical risk prediction
**Transversal**											
	Integration and interaction	Learning model, robotic surgery performance, robotic surgery, robotic surgeon system and, clinical learning	Robotic surgery, robot-assisted laparoscopic prostatectomy, robot system for laparoscopic prostatectomy, cognitive and neural network, and artificial neural oscillator		Robotic network, clinic model, network-controlled system, robotic surgery, and cognitive control		Behavior recognition and prediction, robotic surgery, social interaction, decision-making and neural network model, and sensor detection		Human-robot interaction, object sensor, neural network algorithm, robotic rehabilitation (driving safety), and driving risk		Neural network in robotics, deep learning optimization, efficacy of emerging technologies, autonomous driving, and design of service robot
	Service	Protein classification and medical prediction, clinical technology, neuron responses, surgical decision, and surgical training simulation	Gene expression, clinical decision support, motion segmentation, image analysis, and robotic technology		Gene and cell, health care risk, clinical treatment, clinical decision, and cancer rehabilitation		Gene sequence, mental health literacy, biomarker and MRI, robotic surgery, and image selection and clinical decision		Hospital health care, health care activity, gene expression and brain injury, child care, and cancer treatment		Technology management, medical device regulation, visual recognition network, virtual reality experience and social activity, and medical system
	AI ethics and philosophy	Health information data, robotic surgery, robot-assisted surgery, medical device, and clinical study model	Robotic surgery, information security, robot surgical behavior, drug control, and surgical method analysis		Robotic surgery, laparoscopic surgery, tumor surgery, thyroidectomy, and predictive fuzzy model		Surgery complication, image training, robotic intervention, tumor resection, and robotic hysterectomy		Imaging and surgery, technology and security under COVID-19, drug design, cancer and mental health, and robot assist		Robotic information security, industrial technology integration, neural network segmentation, behavioral learning classification, and medical analysis review

^a^AI: artificial intelligence.

^b^ANN: artificial neural network.

^c^MRI: magnetic resonance imaging.

^d^ECG: electrocardiography.

^e^NLP: natural language processing.

^f^fMRI: functional magnetic resonance imaging.

The recurring presence of specific topics across various domains (as detailed in Table 3) is notable. This convergence can be attributed to articles encompassing multiple topics, with keywords that resonate with >1 domain. For instance, terms related to machine learning, deep learning, and neural networks are evident in 5 distinct domains: reasoning, communication, perception, integration and interaction, as well as AI ethics and philosophy. Although these terms do not appear explicitly in the learning domain, topics from this domain, such as predictive medicine, disease diagnosis, and behavior recognition, are often underpinned by machine learning, deep learning, and neural network methodologies. This absence of explicit terminology might stem from the emphasis of titles and abstracts on application rather than detailing the specific methodology, reflecting variations in thematic focus. The widespread adoption of these techniques across diverse domains indicates their fundamental role in shaping AI applications within medicine [53]. In addition, themes centered on diagnosis and medical applications consistently surface in several domains, underscoring the transformative potential of AI in augmenting diagnostic precision and treatment efficacy.

## Discussion

### Principal Findings

Delving into the realm of AI in medicine, our analysis yielded profound insights across multiple facets. The following is a preliminary snapshot of our key findings:

- Over the past 23 years, the medical evolution of AI has been remarkable, with the United States leading and China quickly catching up. The learning domain is a central focus, which is complemented by growth in areas such as AI ethics.
- Research from the United States stands out in influence, as evidenced by citation counts. Despite China’s large publication volume, Europe, especially the United Kingdom, Germany, and France, shines in impactful contributions. The learning domain dominates in citations owing to its research significance and volume.
- AI research presents distinctive thematic clusters, with certain “hub” publications guiding the direction of AI in medicine.
- LDA-based analyses reveal pivotal roles for machine learning, deep learning, and neural networks within AI disciplines. These findings align with scholarly insights that underscore the transformative role of AI in diverse areas of clinical practice.

### Publication Analysis

Our research has highlighted the swift evolution of AI in medicine, underscored by an accelerating publication rate over the past 23 years [54-61]. The world map of this progression prominently features the United States and China, with both nations leaving a discernible footprint on AI medical research. With its consistent contributions over the last 23 years, the United States has cemented its position as a stalwart, but China’s burgeoning contributions hint at a potential shift in the epicenter of AI-driven medical research in the coming years. The learning domain, as defined by the JRC, emerges as a primary focus in AI research. Keywords within this domain appear frequently, underscoring its central role in the field. Concurrently, other domains such as communication, integration and interaction, and services have witnessed growth, with their share of the overall research staying relatively consistent. Notably, burgeoning areas such as AI ethics and the philosophy of AI demonstrate notable growth, reflecting the expanding boundaries of the application of AI in medicine.

### Citation Analysis

Examining citation counts reveals the influential stature of works predominantly emerging from the United States. These citations not only signify the gravitas of the original research but also mirror the collective acknowledgment by peers and the broader scientific community. Intriguingly, although China demonstrates a robust publication volume, its citation counts remain relatively modest. In contrast, European countries, especially the United Kingdom, Germany, and France, despite having fewer publications than China, have achieved notably higher citation counts, underscoring the impactful nature of their research contributions. Furthermore, when diving into domain-specific data, the learning domain distinctively surges ahead in citations. This is not just a testament to the significance of research within this domain but also reflects the sheer volume of publications related to it, which outpaces other domains considerably.

### Science Mapping

Venturing into the realm of scientific mapping, our study revealed a distinctive segmentation within AI research. Notable thematic clusters, albeit sparsely interlinked, depict specialized and perhaps compartmentalized advancements within specific AI niches. This mapping hints at the emergence of “hub” publications, which are seminal works that, despite their limited number, have profoundly steered the direction of AI in medicine.

### Topic Analysis With Topic Modeling (LDA)

Further deep dives using the LDA method have highlighted particular trajectories within AI disciplines. It is evident that machine learning, deep learning, and neural networks play pivotal roles, appearing recurrently across various AI subdomains. Their prevalence underscores their foundational significance in medical AI applications, particularly in areas such as medical imaging enhancement, which points toward a future of even more accurate diagnostic methods [62]. Another salient observation from our study is the void that exists in terms of a universally accepted definition of AI in medicine. Such a gap emphasizes the need for continued dialogue and consensus building within the academic community.

In juxtaposition with prior studies, our observations echo several scholars’ insights into the medical AI domain. Li et al [62], for instance, have stressed the superiority of AI-integrated imaging. Concurrently, a plethora of researchers, including Sanchez-Martinez et al [63], Filiberto et al [64], Adlung et al [65], and Kim et al [66], have underscored the indispensable role that machine learning now plays in clinical practice and decision-making processes.

### Detailed Domain Insights

#### Overview

Building on our previous insights, our classification analysis delved deeper into the intricacies of each AI research domain. Although specific keywords are evident across multiple domains, their thematic intensities differ, causing topics to be represented differently in various areas. Despite this overlap, each research field unmistakably has its unique focal points. This granularity sheds light on the nuanced topic shifts and specific considerations of AI within each domain of the medical landscape.

#### Reasoning

In the reasoning domain, we found a relatively uniform distribution of articles across the top 5 topics for each period. Diagnosis-related topics accounted for a dominant proportion in the first decade [67-69]. In contrast, the following decade saw a decline in diagnostic-centric research, with an increase in disease prediction and clinical decision support studies [70-74].

#### Planning

Initial research within the planning domain was evenly spread across various topics, including imaging processing [75], artificial neural network algorithm [76], and robot-assisted surgery [77,78], without any particular area dominating. However, a shift occurred between 2010 and 2014, with computer-aided disease detection and prediction taking the lead [79-82]. Recently, there has been a surge in drug development and disease-drug response studies [83,84], with COVID-19–related research dominating the most recent publications [85].

#### Learning

The learning domain houses markedly more research articles than other areas, with an initial focus on learning algorithms [86,87]. Over the years, a pivot occurred toward using AI to enhance medical imaging [88-93], making it the leading topic in this field. This shift from algorithm-centric research to application-based studies is reflected in other AI domains as AI technology matures [94-96]. Despite the dominance of medical imaging, other topics, such as gene and protein sequences, have also received attention [97,98], albeit to a lesser extent.

#### Communication

With the rise of electronic health records in clinical practice, research in the communication domain has primarily focused on semantic analysis [99]. The integration of machine learning has equipped electronic health records with documentation capabilities and decision support functionality [99-101], a shift that our research corroborates.

#### Perception

Research patterns in the perception domain echo those of the planning domain. Initially, a range of topics received equal attention; however, recently, some have taken the lead. Although pattern recognition [102] has been the general direction, images [103], speech [104], sounds [105], and entity recognition [106] have dominated the past decade, with activity reconnection taking the forefront in recent years.

#### Integration and Interaction

The distribution of topics over time within this domain was relatively even. However, robotic research, including robot-assisted surgery [107], robotic surgery evaluation [108], and human-robot interaction [109], have consistently held sway over the past 2 decades.

#### Service

The increasing number of articles in the service domain reflects a shift from an even distribution of topics to a few dominating areas. The initial research was broad, including protein and gene expression [110], clinical techniques [111], and clinical decision support [112,113]. The increase in studies led to genes [114,115] becoming a dominant topic, a shift that occurred again in the past few years, with the focus now on hospital health care and disease treatment [116].

#### AI Ethics and the Philosophy of AI

Research within AI ethics and philosophy predominantly focuses on surgery, particularly robot-assisted surgery, indicative of the nascent stage of this field [117].

#### General Tendencies

Our topic modeling results provide a clear picture of the focus of AI research in medicine over the past 2 decades and allow us to predict potential future directions in this field. Domains that attract a substantial volume of research are likely to continue to influence the direction of medical AI research. The learning domain, primarily machine learning and deep learning, has been and will likely remain at the forefront. Given the growing interest, the domain of AI ethics and philosophy is also anticipated to gain more attention.

Our analysis of topics within each domain further reveals that some medical regions, aided by predictive algorithms, pattern recognition, and image analysis capabilities, will likely gain prominence. Research related to medical diagnostics, robotic interventions, and disease management appears to be future areas of focus [118,119].

### Limitations

Our study has several limitations. First, we based our keyword selection on the JRC AI Watch report owing to the absence of a clear medical AI taxonomy. Consequently, our results included a minimal number of articles on nonmedical research on PubMed, especially in the integration and interaction domain. We opted not to exclude those interdisciplinary interfaces as they contribute to overall AI development.

Second, our LDA topic modeling focused on the titles and abstracts of articles, which may not be as comprehensive as a full-text analysis. Future studies should consider a full-text approach to enhance the results.

Third, our search was limited to English articles, potentially excluding pertinent research in other languages. This suggests a need for future research with varied linguistic contexts.

Finally, the LDA topic modeling function occasionally produced vague combinations of keywords, necessitating the inclusion of a PMID for better topic interpretation. This limitation of the LDA should be considered in future studies.

### Implications

Although some previous studies have used the LDA approach [120,121], our work pioneers a comprehensive and temporal analysis of AI in the medical literature. Theoretically, this study provides a holistic and temporal perspective on AI in medicine. By leveraging LDA for topic modeling in tandem with dictionary-based, computer-assisted text analysis, we have mapped the evolutionary shifts within medical AI research domains. Such an approach advances our current methodology and may serve as a reference point for subsequent research that seeks to trace the developmental arcs of other intricate research areas. Notably, the observed transition from an algorithm-centric to an application-driven research paradigm provides additional layers to the academic dialogue, suggesting a more applied focus for future studies in the domain.

From a practical perspective, our findings have implications for the broader medical community. The increased emphasis on application-based AI research hints at a future where advancements in medical imaging, predictive algorithms, and pattern recognition may become mainstays in clinical settings, offering the potential for enhanced patient outcomes. As these technologies mature, health care practitioners must remain attuned to their developments to ensure that they are harnessed optimally for patient care.

Moreover, the heightened discourse around AI ethics underscores an urgent need for medical professionals and policy makers to internalize and operationalize ethical considerations when implementing AI-driven interventions. As our research elucidates potential growth trajectories, areas such as diagnostics, robotic interventions, and disease management emerge as prominent frontiers. Such insights can provide direction to both clinical practitioners and industry decision makers, helping them navigate the evolving nexus of AI and medicine.

### Conclusions

The transformative potential of AI in medicine is becoming increasingly evident, although the field is still nascent in unlocking its complete range of possibilities. Our analysis offers a holistic snapshot of the existing medical AI research landscape, charting its progression and thematic pivots over time.

Leveraging the power of Python, we meticulously extracted a vast amount of literature from the Entrez database. Through a rigorous analysis of keyword frequencies in titles and abstracts, we stratified our findings into 8 distinct AI domains, as delineated by the JRC AI Watch report. This categorization proved instrumental in bridging the gap left by the absence of a consolidated taxonomy for medical AI.

Our subsequent LDA topic modeling, rooted in the framework of the AI Watch report, unraveled specific thematic threads within each domain. A salient revelation from our study is the discernible shift in medical AI research orientation from an early, intense concentration on AI algorithms to an emphasis on tangible applications. This transition is not only reflective of the maturation of AI technology but also indicative of a burgeoning consciousness about AI’s ethical dimensions. This is further corroborated by the burgeoning volume and significance of research centered on AI’s ethical ramifications each subsequent year.

To encapsulate, this study offers a panoramic view of the multifaceted AI research terrain in medicine, delineating its growth vectors. As we stand at this juncture, the anticipation is for more pronounced tilts toward tangible applications and a heightened ethical discourse, auguring a future for AI in medicine that is both technologically advanced and ethically grounded.

## Appendix

Multimedia Appendix 1 Search strategy.

## Abbreviations

AI: artificial intelligence
JRC: Joint Research Centre
LDA: latent Dirichlet allocation
MeSH: Medical Subject Headings
PMID: PubMed identifier

## Data Availability

The data sets generated or analyzed during this study are available from the corresponding author upon reasonable request.
