# Supplementary material for: Mapping the Bibliometrics Landscape of AI in Medicine: Methodological Study
Source: J Med Internet Res. 2023 Dec 8;25:e45815. doi: 10.2196/45815 (PMC10746970; doi:10.2196/45815)
Supplement: Multimedia Appendix 1 [file jmir_v25i1e45815_app1.docx]

# Search strategy

## Objective

This study aimed to outline the current landscape and future trends in medical AI research, examining all AI-related studies within PubMed over the past two decades.

## Database selection

Our primary source for acquiring pertinent literature is PubMed, a service of the US National Library of Medicine (NLM) [26]. Esteemed by both librarians and researchers, the resources accessible through PubMed from NLM are known for their high quality and dependability [26]. PubMed operates as a search engine for MEDLINE, other NLM biomedical and life science journals, and online books, making it an essential part of the Entrez literature retrieval system [26].

With more than 34 million citations and abstracts [27], PubMed stands as a comprehensive and readily available resource in the biomedical and health domains. Several prior studies have been based exclusively on articles published in PubMed, exemplifying its reliability as a research source. For instance, Boyack et al [28] developed a detailed and precise literature research model solely based on PubMed publications. Similarly, Wang et al [29] built their research on intricate biological relationships using data from over 300,000 articles published in PubMed. Kveler et al [30], too, used computational mining in PubMed to predict and validate cell-cytokine interactions.

Such instances reinforce our belief in conducting research using papers published in PubMed, underlining the credibility and validity of this approach.

## Keywords

Previous research often employs a broad concept of [17,19], conducting the literature reviews based on specific keywords, such as machine learning or deep learning [14,26].

However, this approach overlooks numerous AI-related articles. Given the absence of a predefined framework for this analysis, it was critical to identify search keywords. Thus, our first crucial task involved a two-pronged search strategy: using Medical Subject Headings (MeSH) terms and associated text keywords derived from those MeSH terms. This methodology was adopted to ensure an expansive capture of medical AI articles. For example, when searching for articles on the topic of deep learning, our Python script was configured to perform a comprehensive scan. This involved not only searching for the MeSH tag “deep learning” but also using "deep learning" as a text search keyword in both the title and abstract. Additionally, we incorporated entry terms associated with the "deep learning" MeSH tag into our search criteria. This search strategy has been shown to enhance the efficiency of the literature review [27].

Upon obtaining the search results from this method, we took the analysis a step further by investigating the distribution of research across various AI domains. To accomplish this, we devised a dictionary based on the eight AI domains and associated keywords identified in the 2020 AI Watch report published by the European Commission Joint Research Centre (JRC) [17] (refer to Table 1). This dictionary served as our analytical tool, helping us discern and understand the evolving research trends in the diverse AI sector based on our search results from PubMed.

## Search terms

Our search terms were identified in August 2022. They were extracted from PubMed MeSH tags and the 2020 AI Watch report. A comprehensive list of search terms and counting keywords are provided in Tables 1 and 2.

Table 1. List of search keywords.

Table 2. List of counting keywords.

## Search Strings Construction

Based on the identified MeSH terms and keywords, we constructed a text query for NCBI’s Entrez API that retrieves a list of unique identifiers – PMID – for PubMed entries matching at least one of the MeSH tags or the search terms in the abstract and title. We employed the “OR” operator to ensure entries were retrieved if any MeSH tags or search terms were present. The exact combinations of search terms and the final search strings employed for this study can be found in Table 3.

Table 3. Combinations of search strings.
